# When ubiquitination meets phosphorylation: a systems biology perspective of EGFR/MAPK signalling

**DOI:** 10.1186/1478-811X-11-52

**Published:** 2013-07-31

**Authors:** Lan K Nguyen, Walter Kolch, Boris N Kholodenko

**Affiliations:** 1 Systems Biology Ireland, University College Dublin, Belfield, Dublin 4, Ireland; 2 Conway Institute, University College Dublin, Belfield, Dublin 4, Ireland; 3 School of Medicine and Medical Science, University College Dublin, Belfield, Dublin 4, Ireland

**Keywords:** Ubiquitination, Ubiquitination-phosphorylation crosstalk, Quantitative modelling, Phosphorylation-induced ubiquitination, MAPK signalling

## Abstract

Ubiquitination, the covalent attachment of ubiquitin to target proteins, has emerged as a ubiquitous post-translational modification (PTM) whose function extends far beyond its original role as a tag for protein degradation identified three decades ago. Although sharing parallel properties with phosphorylation, ubiquitination distinguishes itself in important ways. Nevertheless, the interplay and crosstalk between ubiquitination and phosphorylation events have become a recurrent theme in cell signalling regulation. Understanding how these two major PTMs intersect to regulate signal transduction is an important research question. In this review, we first discuss the involvement of ubiquitination in the regulation of the EGF-mediated ERK signalling pathway via the EGF receptor, highlighting the interplay between ubiquitination and phosphorylation in this cancer-implicated system and addressing open questions. The roles of ubiquitination in pathways crosstalking to EGFR/MAPK signalling will then be discussed. In the final part of the review, we demonstrate the rich and versatile dynamics of crosstalk between ubiquitination and phosphorylation by using quantitative modelling and analysis of network motifs commonly observed in cellular processes. We argue that given the overwhelming complexity arising from inter-connected PTMs, a quantitative framework based on systems biology and mathematical modelling is needed to efficiently understand their roles in cell signalling.

## Introduction

Cell signalling crucially depends on a repertoire of posttranslational modification (PTM) mechanisms for its regulation. Protein ubiquitination, the covalent attachment of the short protein modifier ubiquitin to target proteins, has emerged as a prevalent modification utilised by signalling processes to regulate a range of functional behaviours. First recognised as a targeting signal to send proteins to the proteosomal degradation pathway [1], ubiquitination has since been implicated in the non-degradative regulation of a plethora of cellular processes, including signal transduction [2], enzymatic activation [2,3], endocytosis and trafficking [4], chromatin rearrangement [5] and DNA repair [6].

**Figure 1:**
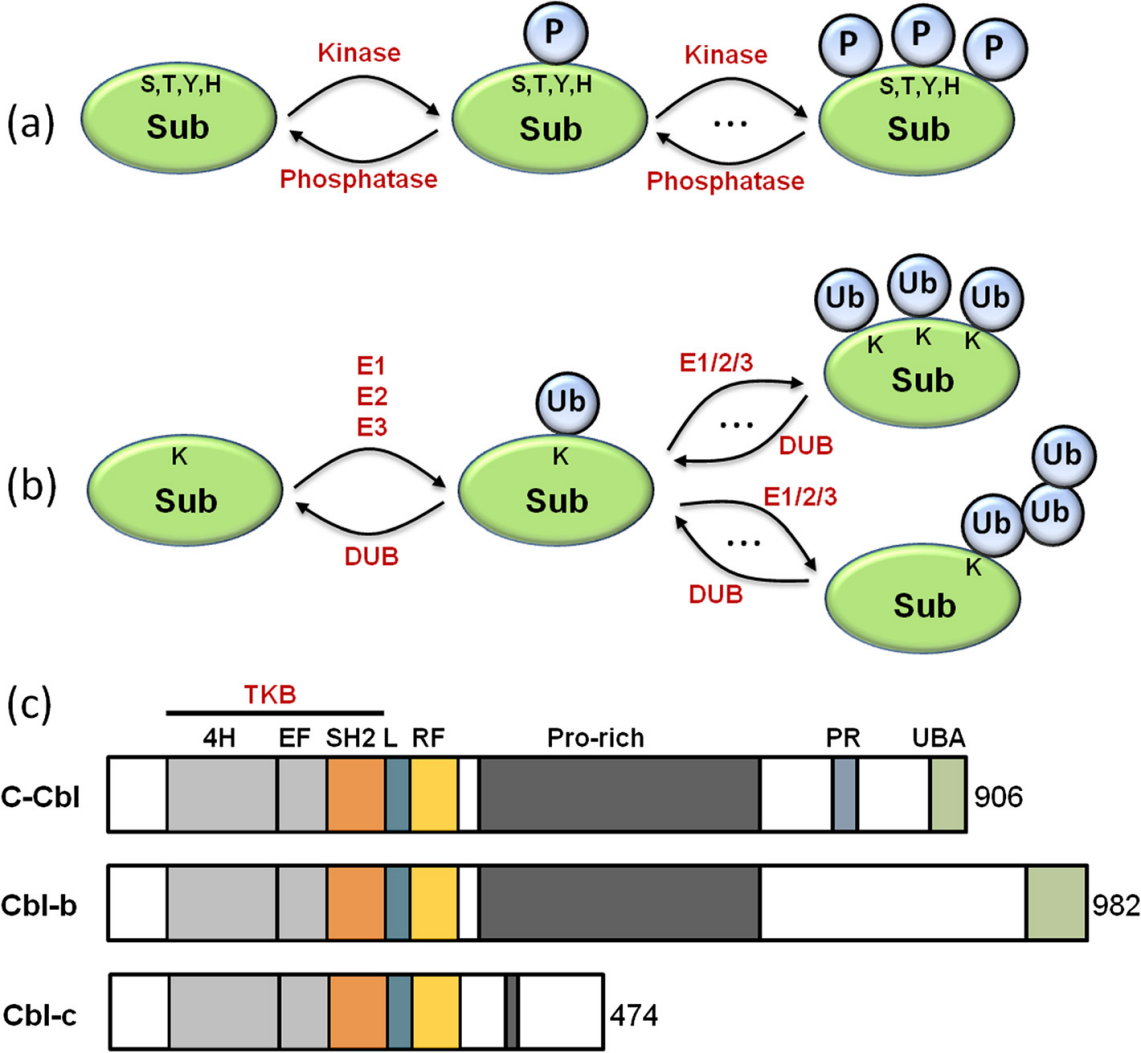
**Illustration of phosphorylation, ubiquitination as PTMs on a substrate, and domain structures of the Cbl protein family. (a, b)** Phosphorylation versus ubiquitination as post-translational modifying mechanisms of a protein substrate. **(c)** Mammalian Cbl protein family domain structures. The Cbl proteins contain, from N to C terminus, a TKB domain, a linker region (L), RING finger domain (RF), Pro-rich regions, poly-Pro-Arg motif (PR) and a UBA domain. The TKB domain consists of a four-helix bundle (4H), an EF hand, and a variant Src homology region 2 (SH2) domain. Cbl-3 lacks the PR and UBA domain.

Unlike phosphorylation where the addition of the phosphate group to the modified targets is a rather straightforward single step, ubiquitination occurs in a three-step reaction requiring three different enzymes: an ubiquitin-activating enzyme (E1), an ubiquitin conjugating enzyme (E2), and an ubiquitin ligase enzyme (E3). Ubiquitin is first activated by E1, followed by conjugation to an E2 before finally ligated to the lysine residues of target proteins by the E3 ligase (Figure 1a,b) [1]. While phosphorylation can occur on several different amino acids, primarily serine, threonine, tyrosine and histidine, only a single phosphate group can be added to a particular residue. In contrast, ubiquitination can only target a single amino acid, i.e. lysine, but can attach multiple ubiquitin residues which can be linked via different types of bonds through any one of the seven lysine residues of the ubiquitin molecule., e.g. monoubiquitination, multi-monoubiquitination, and polyubiquitin chains (Figure 1a,b). The versatile diversity of signalling associated with ubiquitination further stems from the myriad ways in which the polyubiquitin chains can be formed, either as uniform (e.g. containing only Lysine 48 or 63 linkages) or as recently discovered atypical branched chains with mixed linkages (e.g. Lysine 6/27/48-linked chains [7]), which seem to serve distinct context-specific functions. Thus like phosphorylation, ubiquitination is a dynamic modification that not only targets proteins for degradation, but can change the conformation and activity of the target proteins. Furthermore, similar to protein phosphorylation, ubiquitination is regulated by pairs of opposing modifying enzymes: E3 ligases and de-ubiquitinating enzymes (DUBs). These regulating proteins, in an analogous manner to kinases and phosphatases, serve to fine-tune the levels of the target protein ubiquitination. An extra level of analogy comes from the observation that, just as the phosphorylation network in which the kinases and phosphatases are often (de)activated by phosphorylation, ubiquitinating enzymes appear to be regulated by ubiquitination events.

Over the past few years, the interplay between ubiquitination and phosphorylation has emerged as a prominent posttranslational crosstalk and a key principle in eukaryotic cell signalling [8]. Phosphorylation often serves as a marker that triggers subsequent ubiquitination, in particular where ubiquitination leads to degradation [9-11]. In many cases, phosphorylation of substrate E3 ligases acts as a signal that can dramatically influence their activity. In other cases, ubiquitination provide a switching mechanism that can turn on/off the kinase activity of certain proteins [12]. Understanding of how these two major PTMs interact to regulate signal transduction is an important topic in cell signalling. In this review, we discuss the involvement of ubiquitination in the regulation of the epidermal growth factor (EGF)-mediated extracellular signal-regulated kinase (ERK) signalling pathway via the EGF receptor (EGFR), and highlight the interplay between ubiquitination and phosphorylation in this system, which beyond its many physiological functions is also a major player in human cancer. The review contains two parts. In the first part we survey recent biological findings related to ubiquitination and crosstalk with phosphorylation as means for the functional control of the components of the EGFR-mediated ERK pathway, and highlight some remaining open questions. In the second part, we demonstrate the rich and versatile dynamics of crosstalk between ubiquitination and phosphorylation by using quantitative modelling and analysis of various network motifs where such crosstalk is often observed. Multiple lines of evidence from both theoretical and experimental studies have shown that intricate dynamics including bistable switches, mutistability and sustained oscillation can be brought about as a result of the interplay between feedback regulations and nonlinear post-translational modification cascades, such as phosphorylation [13-16], ubiquitination [3] and GTPase cascades [17]. Oscillations in GTPase cascades drive periodic protrusion and retraction of lamellipodia during cell migration [18,19]. In addition, short-period (20 min) and long-period (4–5 hrs) ERK oscillations have been experimentally reported [15,16]. It is likely that these complex dynamics may also emerge from crosstalk between phosphorylation and ubiquitination. Our aim here is to illuminate non-trivial dynamics arising from these generic crosstalk mechanisms that would apply not only to the EGFR pathway but to many other pathways. We argue that given the overwhelming complexity originating from interconnected PTMs, a quantitative framework based on systems biology and mathematical modelling is needed to efficiently understand their regulatory roles in cell signalling [20].

### Involvement of ubiquitination in EGFR-mediated MAPK signalling pathway

#### Ubiquitin-mediated regulation of EGFR, adaptor proteins and roles in endocytosis

The function of ubiquitination as a regulatory mechanism in Receptor Tyrosine Kinases (RTKs) endocytosis was one of the early findings of the non-proteolytic roles of this PTM in cell signalling [21,22]. Ubiquitination of the receptor and endocytic adaptor proteins was found critically important in mediating EGFR internalisation and downstream signal transduction. The proteins of the Cbl family, consisting of three mammalian homologs c-Cbl, Cbl-b and Cbl-3, are the best characterized E3 ligases that regulate the EGFR endocytosis pathway. Located next to the RING finger domain, which is responsible for transferring ubiquitin to substrates, the Cbl N-terminal region is composed of three conserved domains: a 4 helix bundle domain (4H), an EF hand-like domain, and a SH2-like domain (Figure 1c). Together, these conserved regions form the TKB (tyrosine kinase binding) domain that enables Cbl to recognise phosphotyrosine residues and interact with phosphotyrosine-containing proteins. Following ligand binding and activation of EGFR by autophosphorylation, Clb directly binds to activated EGFR via the TKB domain [23-25]. Cbl can also be recruited to activated RTKs through its constitutive binding partner Grb2 which directly binds to RTK phosphotyrosines via its SH2 domain [26-28]. Recent structural studies suggested that once bound, Cbl becomes phosphorylated on a critical tyrosine (371 in c-Cbl and 363 in Cbl-b) due to the opening-up of the compact structure within Cbl that previously hides the E2 binding site [29,30]. This phosphorylation enables full rotation of the Cbl linker region which exposes the RING domain enabling binding of the ubiquitin-loaded E2 complex. This then triggers allosteric activation of the E2 and stimulates Cbl E3 ligase activity resulting in the subsequent multi-monoubiquitination and polyubiquitination of the EGFR [29,30].

Ubiquitination-related mechanisms regulating the adaptor proteins also play crucial roles in the functioning of the endocytotic pathway, including cargo recognition and delivering. These adaptors include proteins at the plasma membrane including the clathrin coat, the EGFR substrate 15 (EPS15), a member of the EPS15-interacting protein family (EPSIN1–EPSIN3), and hepatocyte growth factor-regulated Tyr kinase substrate (HRS) at the endosomes. Adaptor proteins, which contain ubiquitin binding domains (UBD) such as the ubiquitin-interacting motif (UIM) can recognise the ubiquitin molecules on the ubiquitinated EGFR. This leads to the assembly of active receptors in clathrin-coated pits of the plasma membrane, endosomes and the multivesicular bodies (MVBs) [31]. Adaptor proteins also undergo ubiquitination upon ligand stimulation through a process known as coupled monoubiquitination, which requires the presence of an intact UBD [32]. For instance, upon EGF stimulation EPS15 interacts directly with NEDD4 via its UBD and is ubiquitinated by NEDD4, a homologous to the E6AP carboxyl terminus (HECT) E3 ligase. NEDD4 then transfers the thiolester-conjugated ubiquitin from its catalytic cysteine residue to the adaptor protein, inducing monoubiquitination [32]. This directs progression of the ubiquitinated receptors toward lysosomal degradation through the ESCRT complexes [31,33].

Ubiquitin-mediated EGFR endocytosis affects the signalling dynamics of the downstream pathways, thereby modulating the cellular decisions. Cells have evolved ways to reverse ubiquitination events through de-ubiquitinating enzymes [34]. The STAM-binding protein (STAMBP, also known as AMSH) is a DUB specifically cleaving the lysine 63 and 48-linked ubiquitin chains anchored at the endosome via interaction with the clathrin coat [35]. Thus, STAMBP counteracts the ubiquitin-dependent sorting of receptors to lysosomes [36]. Another DUB which can abrogate the endocytosis of EGFR receptors is USP8 [37]. Before being incorporated into internal vesicles of MVBs, the ubiquitinated EGFR can undergo USP8-induced deubiquitination which moves the EGFR into the recycling pathway back to the plasma membrane [38]. Interestingly, USP8 can be tyrosine and serine phosphorylated in an EGFR- and Src-kinase dependent manner [39]. Since decreased USP8 tyrosine phosphorylation is associated with enhanced endosomal recycling of EGFR when cells are stimulated by TGFα, it is likely that USP8 phosphorylation may regulate its DUB activity. Further research is required to shed more light on this issue.

#### Ubiquitin-mediated regulation of Ras as a major EGFR effector

**Figure 2:**
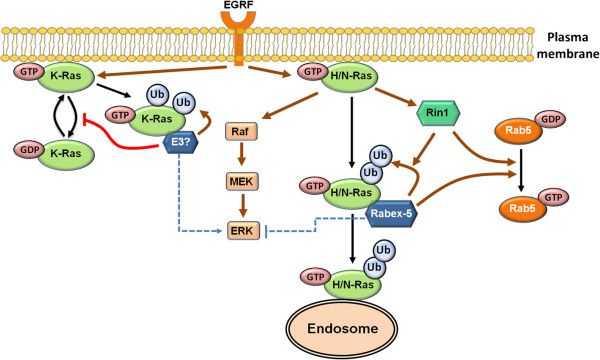
**Schematic representation of ubiquitination-mediated action of Ras isoforms.** H/N-Ras mono- and di-ubiquitination controlled by Rabex-5 promotes their endosomal association, leading to the attenuation of Ras-ERK signalling. On the other hand, K-Ras mono-ubiquitination catalysed by a yet unknown E3 ligase inhibits its GAP-mediated hydrolysis, leading to an increase in its GTP-bound active form and strengthening Ras-ERK signalling. Both Rin1 and Rabex-5 are GEFs for the GTPase Rab5, however the GEF activity of Rabex-5 is not required for ubiquitinating H/N-Ras, while Rin1 GEF activity is necessary for enhancing Rabex5-mediated ubiquitination of the H/N-Ras isoforms. Gray arrows indicate catalysis, black arrows indicate transformation and red blunt arrows indicate inhibition.

Ras is a small GTPase that connects RTK activation to the triggering of many downstream effector pathways including MAP kinase cascades. Ras exists in three isoforms: H-Ras, N-Ras and K-Ras which, despite sharing some regulators and effectors due to similar interaction domains, exhibit divergent functional properties and involvement in carcinogenesis. In certain cell types, K-Ras is the most potent activator of Raf-1 [40,41], whereas H-Ras most efficiently activates PI3K [40]. K-Ras is frequently activated by mutations in cancers of the lung, colon, pancreas and biliary tract, whereas activated mutations of H-Ras and N-Ras are much rarer and mainly confined to urinary tract tumours in the case of H-Ras, and leukemia, melanoma and neuroblastoma in the case of N-Ras [42]. These observations beg the question which biological mechanisms govern the functional differences among the Ras isoforms. A major contributor to functional diversification seems to stem from the differential localisation of the Ras isoforms. Ras subcellular localisation is mainly determined by the fatty acid (farnesylation and palmitoylation) modifications of the C-terminus and the amino acid sequence of the adjacent hypervariable region. However, ubiquitination is an important dynamic modifier of localisation. In a seminal study, Jura et al. showed that H-Ras (and N-Ras), but not K-Ras, are subject to ubiquitination in the Chinese hamster ovary CHOK1 cells. Ubiquitination subsequently promotes the association of H- and N-Ras with the endosomes, thereby modulating the capacity to activate the Raf/ERK pathway (Figure 2) [43]. An H-Ras mutant incapable of being ubiquitinated is a 4-time stronger activator of ERK than the wild-type, suggesting that H-Ras ubiquitination impairs ERK signalling. Ubiquitin conjugation of H-Ras was found to occur mainly by mono- and di-ubiquitination on Lysine 63, with diubiquitin conjugates being the more predominant species [43]. Interestingly, H-Ras ubiquitination was constitutive and not affected by EGF treatment or H-Ras activity state, but seems to depend on the H-Ras hyper-variable region located at the C-terminus [43,44]. Consistent with these results, maintaining a certain level of Ras ubiquitination is vital to prevent inappropriate Ras/ERK activation in Drosophila [45].

In an effort to identify the molecular mechanism by which Ras ubiquitination is regulated, Xu et al. found that Rabex-5 (Rab5 GDP/GTP exchange factor), known previously as a GEF for Rab5 [46,47], is also an E3 ligase for H- and N-Ras [48]. This discovery was supported by the earlier knowledge that Rabex-5 possesses a zinc finger (ZnF) domain similar to that of A20 with E3 ligase activity [49-51], and that Rabex-5 interacts with Ras [52,53]. Using *in vivo* and *in vitro* ubiquitination assays along with RNAi technology, the authors showed that Rabex-5 is necessary and sufficient to catalyse H/N-Ras ubiquitination, promoting their endosomal localisation and resulting in suppressed ERK activation (Figure 2) [48]. Overexpression of Rabex-5 did not induce K-Ras ubiquitination, suggesting Rabex-5 is specific to H/N-Ras. Importantly, a mutation in the ZnF domain but not the GEF domain blocked Rabex-5’s ability to ubiquitinate Ras, indicating that Rabex-5 GEF activity is not required for ubiquitination. Interestingly, this is not the case for Rin1, which is a Rab5-directed GEF, where the GEF function is required for enhancing Rabex-5-dependent Ras ubiquitination (Figure 2) [48]. Because Rin1 is a Ras effector [54], this constitutes a negative feedback which serves to attenuate Ras-mediated ERK signalling. This mechanism is consistent with earlier observations that Rin1 competes with Raf-1 for binding to Ras [54,55]. What remains unclear is how these distinct mechanisms of diminishing ERK signalling interplay at specific cell locations. Adding to the already complex picture, Rabex-5 was known to undergo coupled monoubiquitination [56], determined by its ability to bind ubiquitin through two independent ubiquitin binding domains (UBDs) [49,51]. However, what is the function of this autoubiquitination and how it is involved in Ras ubiquitination are open questions.

Although the studies by the Bar-Sagi group [48,57,58] did not find ubiquitination of K-Ras, it has been reported that K-Ras could be monoubiquitinated in HEK293T cells, preferably at lysine 147 [59]. These discrepancies are most likely due to the usage of different cell types, which may differ in the expression of E3 ligases or the DUBs which determine the detectable levels of K-Ras ubiquitination. Interestingly, the ubiquitination of K-Ras strongly enhances ERK signalling as opposed to H-Ras ubiquitination, indicating dramatic isoform-specific functional difference. Monoubiquitination of K-Ras results in its enhanced GTP loading, whereas for the oncogenic G12V-K-Ras mutant, monoubiquitination increases Ras binding to its main downstream effectors including Raf-1 and PI3K [59]. In identifying the molecular mechanism responsible for the monoubiquitination-mediated activation of K-Ras, Baker et al. recently showed that monoubiquitination at lysine 147 does not alter K-Ras’s intrinsic biochemical properties, but strongly inhibits GAPs-mediated hydrolysis resulting in increased GTP-bound population of monoubiquitinated Ras *in vivo*[60]. Combined, these findings illuminate a novel role for ubiquitin in controlling Ras activity, in addition to regulating its spatial location. It however remains to be discovered whether a similar regulatory mechanism exists for other Ras isoforms under other cellular contexts. It is also noteworthy that all Ras isoforms are subject to polyubiquitination mediated by the F-box protein b-TrCP (b-transducin repeat–containing protein), leading to proteasome-dependent degradation of Ras [61]. In conclusion, the above studies suggest that ubiquitination is an essential mechanism controlling Ras compartmentalisation and its signalling output.

#### Ubiquitin-mediated regulation of components of the Raf/MEK/ERK MAPK cascade

The transduction of a cellular signal as it propagates through the MAPK cascades, exemplified by the Raf/MEK/ERK module, is predominantly controlled by phosphorylation events where typically, each kinase in the cascade is activated by an upstream kinase and inactivated by relevant phosphatases. However, accumulating evidence has revealed that components of this cascade also can undergo ubiquitination, which not only leads to the degradation of the substrate proteins but also appears to regulate their activity and/or localisation [62].

Raf proteins are the main effectors of Ras [63,64] and direct activators of MEK [65,66], serving as essential connectors linking Ras to the MEK-ERK pathway. Extensive work focusing on Raf regulation have revealed a complex, yet still incomplete, picture of the Raf activation/inactivation cycle where phosphorylation events play major regulatory roles (reviewed in [67]). In contrast, the involvement of ubiquitination in the modulation of Raf has received far less attention and remains largely elusive. Raf-1 exists in a complex with the heat shock protein HSP90 and this association is essential for Raf-1 stability [68]. Using NIH3T3 cells treated with GA (the benzoquinone ansamycin Geldanamycin) to disrupt the Raf-1-HSP90 complex which induces rapid Raf-1 degradation, Schulte et al. [69] then used different inhibitors for various proteolytic systems to investigate the mechanisms responsible for the degradation of Raf-1. Inhibition of the proteosome, rather than of the lysosome or other proteases, prevented the observed enhanced Raf-1 degradation. Moreover, the Raf-1 fraction protected from GA-induced degradation showed a smearing pattern typical of polyubiquitinated proteins [69]. These data indicate that Raf degradation involves ubiquitination and the proteosome-mediated pathway. The next important question emerges as to how Raf’s proteosomal degradation is regulated. Investigating if the kinase activity of Raf-1 is regulating its degradation, Noble et al. argued that that Raf-1 kinase activity is required to induce an (in cis) autophosphorylation of the site S621 which helps stabilise Raf-1 [70]. Interestingly, autophosphorylation does not appear to regulate B-Raf stability, since the equivalent S729 site is not autophosphorylated in B-Raf, and B-Raf activity has no effect on its expression level [70]. Clearly, additional work must be done to further elucidate the Raf ubiquitination-related regulation.

Although evidence pointing to an ubiquitination-related mechanism involving MEK in mammalian cells is sparse, the yeast MEK protein Ste7 has been shown by multiple studies to undergo ubiquitination and regulate MAPK specificity [71-73]. The terminal kinases of the cascade, ERK1 and ERK2 have been shown to be ubiquitinated by MEKK1, a MAP kinase kinase of the STE11 family [74]. MEKK1 phosphorylates several MEKs, and its major targets are MKK3 and MKK4, which in turn activates JNK [75,76]. In addition to activating JNK, MEKK1 is also known to regulate ERK signalling [77]. Lu et al. showed that MEKK1 has a dual role as a kinase that also has E3 ligase activity due to a separate kinase domain and a RING-finger like structure containing the PHD domain [74]. Under stress stimulation induced by sorbitol, MEKK1 directly interacts with and poly-ubiquitinates ERK1/2, sending it for degradation which subsequently leads to down-regulation of ERK activity. This however is not the case for serum or EGF stimulation [74]. The dual role of MEKK1 appears to provide opposing controls over ERK, with activating function and also inhibiting function as a direct de-stabiliser. It is important though to note that the existence of multiple regulatory mechanisms does not necessarily imply that they are simultaneously active, but one may be favoured over another under certain physiological conditions. Interestingly, the MEKK1 kinase activity was found to be involved in ERK1/2 ubiquitination [74]. Furthermore, MEKK1 undergoes non-proteolytic self-ubiquitination which inhibits its catalytic activity as a kinase, attenuating MEKK1-mediated phosphorylation of MKK3/4 and resulting in inhibition of ERK1/2 signalling [12]. This represents a rather interesting case where ubiquitination modifies the kinase activity rather than ligase activity of the modified protein. A recent study further reported that under hyperosmotic stress, another MAPK kinase kinase, MEKK2, mediates the transient activation of ERK [78]. However, unlike MEKK1, MEKK2 is instead controlled by an external E3 ligase, the carboxyl terminus of Hsc70-interacting protein (CHIP). CHIP depletion attenuates the degradation of MEKK2 and prolongs ERK activity.

#### Roles of ubiquitination in crosstalked pathways

##### Functional roles of Itch in the EGFR/ERK signalling pathway

ITCH is the HECT E3 ubiquitin ligase belonging to the NEDD4 protein family. It is characterised by the N-terminal C2 domain responsible for membrane localisation, 2 to 4 WW domains involved in substrate recognition, and the C-terminal catalytic HECT ligase domain [79]. Although ITCH is better known for its role in the immune system development [80,81] where its deficiency causes syndromic multisystem autoimmune disease [82], increasing evidence implicates ITCH involvement in EGF signalling and EGF-mediated anti-apoptosis.

##### ITCH self-ubiquitination increases its activity

**Figure 3:**
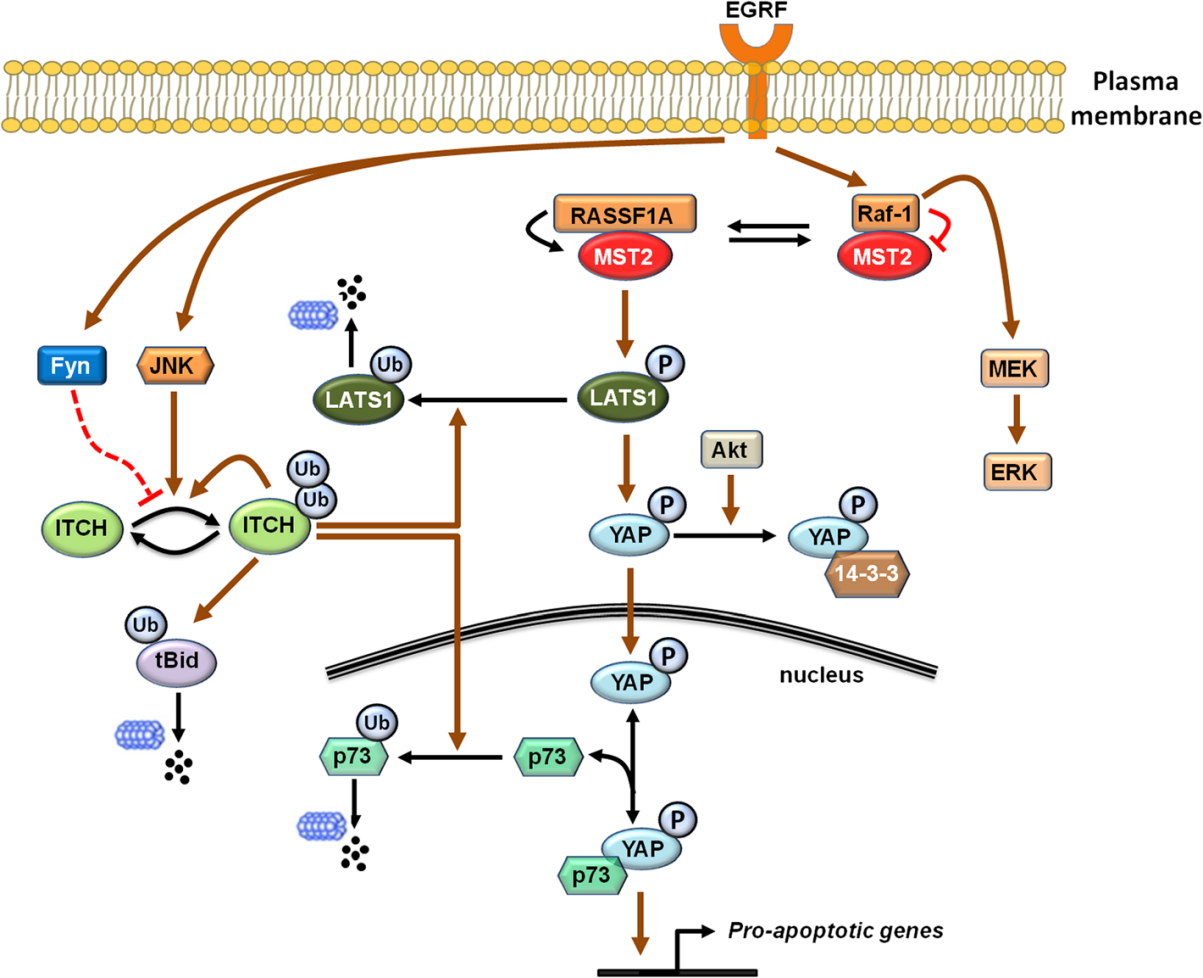
**Schematic representation of ITCH self-ubiquitination and its involvement in crosstalk between Raf/MEK/ERK and Raf/MST2/LATS1/YAP signalling.** Ubiquitin ligase activity of ITCH is negatively regulated by Fyn-mediated tyrosine phosphorylation but positively by JNK-mediated serine/threonine phosphorylation. The MST2/LATS1/YAP signalling cascade is triggered by RASSF1A as a result of a balancing act between RASSF1A-MST2 and MST2-Raf-1 complexes. Akt-mediated phosphorylation of YAP leads to its sequestration by 14-3-3. Active YAP translocated into the nucleus binds p73 to induce pro-apoptotic gene expression. Active Itch ubiquitinates and promotes proteosomal degradation of tBid. Itch also poly-ubiquitinates LATS1 and p73 and targets these proteins for degradation. Gray arrows indicate catalysis, black arrows indicate transformation and red blunt arrows indicate inhibition.

ITCH can catalyse its own ubiquitination. However, the self-ubiquitinated conjugates of ITCH do not have K48-linked polyubiquitin chains, which would target the protein for degradation like most other E3 ligases. Instead they have K63 linkages, which serve to promote ITCH ligase activity [83], establishing a non-degradative role for ITCH self-ubiquitination (Figure 3). Importantly, ITCH self-ubiquitination follows an intermolecular interaction mechanism rather than intramolecular reactions [83]. It has been recognised that intermolecular self-modification (including phosphorylation and ubiquitination) can induce complex dynamic behaviours including bistability, multistability, sustained oscillations and excitability [3,13]. Subsequent reports further identified JNK as the upstream kinase of ITCH. JNK-mediated phosphorylation promotes ITCH self-ubiquitination and greatly stimulates ITCH activity [84,85] (Figure 3). Phosphorylation of three sites, S199, S232 and T222, located within a proline-rich region of ITCH is necessary and sufficient to disrupt an inhibitory interaction between the WW and HECT domains of ITCH, triggering a conformational change that boosts the catalytic activity of its ligase function [84]. Furthermore, treatment of cells with EGF leads to JNK-dependent phosphorylation of ITCH, stimulating its activity [85].

##### ITCH connects EGF signalling and apoptotic pathway

ITCH was demonstrated to interact with the truncated form of the proapototic protein Bid (tBid), ubiquitinate tBid and induce its proteosomal degradation [86] (Figure 3). tBid is a truncated form of Bid arising through caspase mediated cleavage during apoptosis. tBid amplifies the mitochondrial apoptosis pathway by binding to and inactivating Bcl2 family proteins promoting mitochondrial permeability transition and apoptosis [87]. In contrast, the full-length form of Bid does not interact with ITCH and is not subject to proteosomal degradation regardless of whether or not ITCH is present [88]. Importantly, the ITCH-mediated down-regulation of tBid increased following EGF treatment [86]. Furthermore, ITCH expression can significantly reduce cell apoptosis induced by tBid and influences the balance between cell survival and apoptosis in normal cell culture conditions [86]. Taken together, these studies suggest a sequence of events involving ITCH that is initiated from the cell surface following EGF treatment: EGF triggers receptor activation which stimulates ITCH auto-ubiquitination partly due to EGF-mediated JNK phosphorylation. This leads to increased degradation of ITCH substrates, including truncated tBid, resulting in decreased apoptosis and thus promoting cell survival.

##### ITCH connects EGF signalling to apoptosis via the MST2 pathway

Another route through which ITCH links EGFR/Raf/ERK signalling to apoptosis is via the MST2/LATS1 pathway (Figure 3). Our group has shown that Raf-1 controls the proapoptotic kinase MST2 activity and restrains cell apoptosis via the Raf-1-MST2 complex formation, which occurs in two ways [89,90]. First, Raf-1 binding interferes with MST2 dimerisation and subsequent activating autophosphorylation [90]. Second, Raf-1 recruits a phosphatase that dephosphorylates the activating sites on MST2, thereby limit its activation [89]. Furthermore, using a signalling pathway mapping strategy based on tracking dynamically changing protein interactions, we have mapped a multistep pathway from the cell membrane through MST2 activation to p73 dependent transcription in the nucleus, in which MST2 directly activates LATS1 [91]. Interestingly, ITCH has been recently reported as an E3 ligase for LATS1 as well as for p73, which targets these proteins for poly-ubiquitination and degradation [92,93]. Thus, the involvement of ITCH as a degradation regulator of key components of the proapoptotic MST2/p73 pathway may link EGF signalling to apoptosis in a manner independent of the Raf-1-MST2 binding (Figure 3). It would be interesting in future studies to explore the role of ITCH in regulating apoptosis in this direction. Furthermore, since ITCH contains a consensus phosphorylation motif for LATS1 substrates, ITCH may be a substrate of LATS1 [92]. Understanding if LATS1 phosphorylates and alters ITCH activity therefore would be an interesting research avenue.

##### Fyn phosphorylation negatively regulates ITCH function

JNK is not the only kinase identified so far to target ITCH. Previous studies has indicated that Src-family tyrosine kinases are targeted for degradation by HECT-domain E3 ligase. Yang et al. therefore set out to investigate whether the Src-family kinase Fyn is ubiquitinated by ITCH in T cells, but instead discovered that ITCH is a substrate for Fyn [94]. Fyn phosphorylates ITCH at Y371 located in the third WW domain. Importantly, an ITCH mutant where Y371 is replaced by phenylalanine causes a substantial increase in association of ITCH and one of its major substrate, JunB [94]. Thus, the ubiquitin ligase activity of ITCH is regulated negatively by Fyn-mediated tyrosine phosphorylation and positively by JNK-mediated serine/threonine phosphorylation (Figure 3). Furthermore, Yang et al. found that ITCH Y371 to Phe mutation did not alter the self-ligase activity of ITCH in T cells, and hypothesised that Y371 phosphorylation results in a structural hindrance for JunB interaction. However, it remains unclear whether this tyrosine phosphorylation would affect the K63 self-ubiquitination of ITCH in other cell lines, such as HEK293, or whether it would affect Bid degradation and tBid-directed apoptosis. It is also open for investigation as to what are the inputs upstream of Fyn which triggers ITCH tyrosine phosphorylation. Nevertheless, it is intriguing to observe a signalling paradigm where two functionally opposing kinases act on a common E3 ligase to tune its activity. We anticipate this paradigm will become more commonly seen as more studies are carried out.

### Quantitative modelling as a tool for analysis of ubiquitination-phosphorylation crosstalk networks

The last decade has witnessed an unprecedented explosion of biological knowledge and large data sets acquired for many signalling processes at the cellular level, largely due to the development of sophisticated and high-throughput biochemical techniques in proteomics and other omics. As part of this trend, the studies reviewed in the previous section, although still limited, have revealed a rather complex picture of how ubiquitination and phosphorylation interplay to regulate signal transduction pathways such as the EGFR. The huge complexity hampers our ability to interpret and predict the regulation of the network as a whole, which is essential to better understand EGFR signalling and its role in diseases. To unravel this complexity and obtain a systems-level understanding of network signalling, systems biology approaches employing quantitative frameworks in forms of mathematical and computational models are emerging as promising solutions. These mathematical models provide a platform for the description, prediction and understanding of the various regulatory mechanisms in a quantitative and integrative way [95-98]. In this section, we describe the rich and versatile dynamics of crosstalks between ubiquitination and phosphorylation by using mathematical modelling to analyse a number of network motifs largely motivated by the biological findings discussed in previous sections, and are commonly seen in other signalling processes besides the EGFR pathway.

#### Phosphorylation-mediated ubiquitination

**Figure 4:**
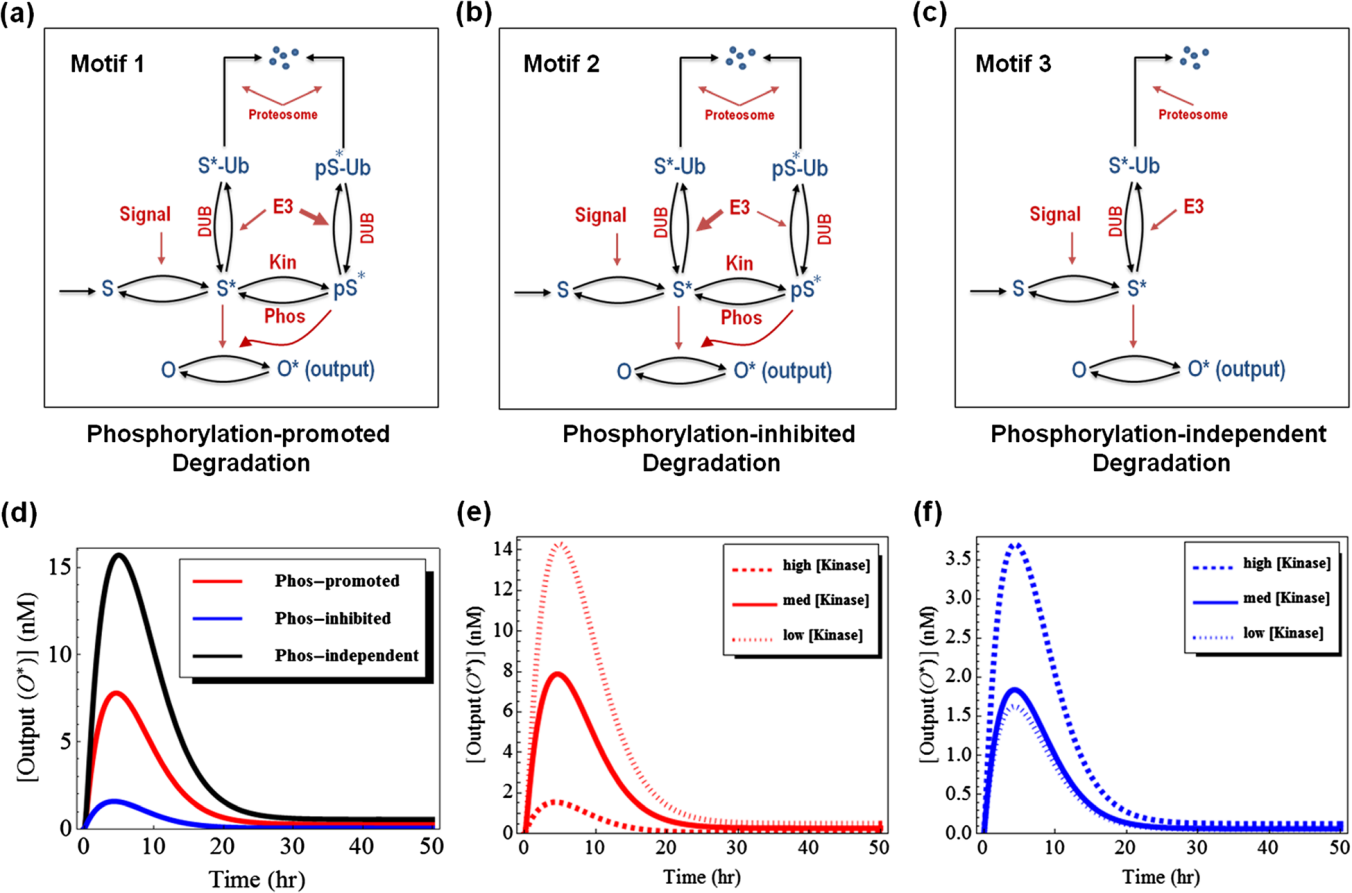
**Kinetic schemes and model simulations for motifs 1–3. (a-c)** Schematic kinetic diagrams of the network motifs 1–3 described in the text. **(d)** Comparative temporal dynamics of the active output level for the three motifs **(e, f)** Comparative temporal dynamics of the active output at increasing Kinase abundance for motif 1 and 2, respectively. Parameter values used: high [Kinase] = 1000 nM, medium [Kinase] = 100 nM, low [Kinase] = 10 nM. Detailed description of the models is given in the Additional file 1 (SI) document, along with the remaining parameter values.

A recurring theme in the interplay between phosphorylation and ubiquitination is that phosphorylation often influences the ubiquitination and thus degradation of the modified protein, such as in the case of c-Myc [99,100], androgen receptor [101] or the yeast transcriptional factor Rpn4 [102]. We consider two motifs where phosphorylation either promotes or inhibits ubiquitination-triggered degradation (named motifs 1 and 2, respectively, and illustrated in Figure 4a, b). Then, we compare these two motifs to a network motif where (de)ubiquitination is not influenced by phosphorylation events, and phosphorylation is omitted (motif 3, in Figure 4c). As shown in the schematic interactions diagrams, a substrate protein S is assumed to be first activated by an input signal to become active S*, which can be phosphorylated by a kinase (Kin) to form pS*, which is dephosphorylated by a phosphatase (Phos). Both S* and its phosphorylated form pS* are ubiquitinated by an E3 ligase (E3) and subsequently targeted to proteosomal degradation. The rate of ubiquitination is much greater for pS* compared to S* in the phosphorylation-promoted degradation motif 1 (Figure 4a), whereas it is much less in the phosphorylation-inhibited degradation motif 2 (Figure 4b). On the other hand, if phosphorylation does not change the (de)ubiquitination and degradation rates as in motif 3 (Figure 4c), it is sufficient to consider the (de)ubiquitination of S* only. In all three motifs, S is constitutively synthesised to allow for a nonzero steady state. For convenience, we assume that both S* and pS* have the same catalytic activities toward a substrate O whose active state (O*) is used as an output of the systems.

**Figure 5:**
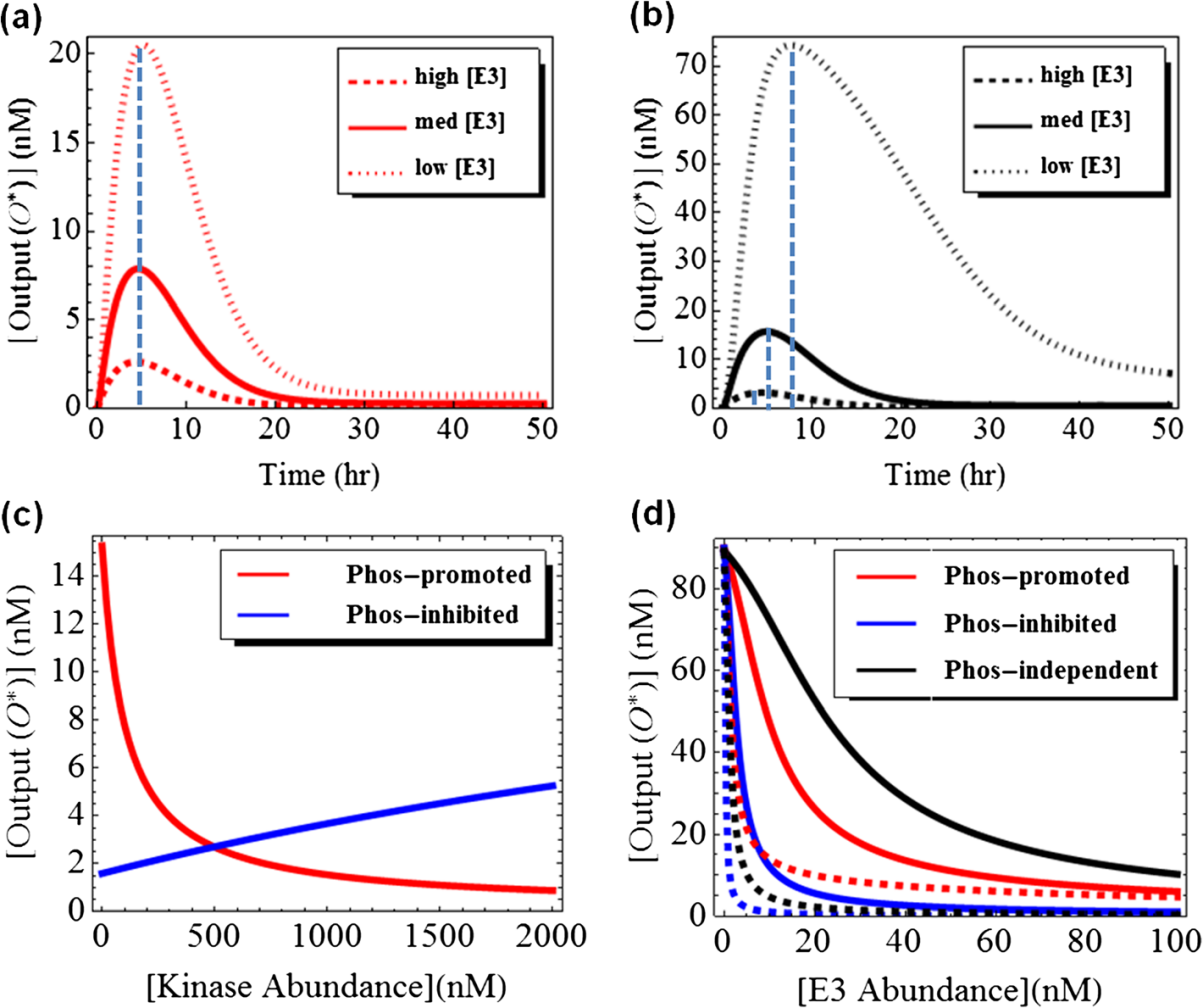
**Model simulations of time-course and dose–response curves for motifs 1–3. (a,b)** Comparative temporal dynamics of the active output at increasing E3 ligase abundance for motif 1 and 3, respectively. Parameter values used: high [E3] = 1000 nM, medium [E3] = 100 nM, low [E3] = 10 nM, the remaining parameters are given in the SI. **(c)** Steady-state dependence of the active output on the Kinase abundance compared for motifs 1 and 2. **(d)** Steady-state dependence of the active output on the E3 ligase abundance compared for three motifs 1–3.

Despite the simplicity of these motifs, intuitive predictions regarding dynamical behaviour of the network components at various abundances of the regulatory proteins (e.g. Kin, Phos or E3) would be a nontrivial task without the employment of mathematical models. We thus constructed models based on ordinary differential equations (ODEs) and the law of enzyme kinetics for these motifs, whose details are given in the Additional file 1 (SI). Using the constructed models, we can simulate time-course as well as steady-state dose–response simulations under various conditions. Figure 4d compares the time-course dynamics following a step-function input signal for the three motifs. Using the parameters of motif 1 as the reference set, the output shows similar transient pattern with similar peak time but different peak values among the compared motifs, with highest peak in motif 3 followed by motif 1 and then 2. This suggests that tuning differential ubiquitination between the unphosphorylated and phosphorylated forms of S by varying the kinase would be a way to modulate the peak of the output without affecting its dynamical form. Indeed, increasing the kinase abundance decreases the output in motif 1 (Figure 4e) and increases the output in motif 2 (Figure 4f) but does not affect the peak time and the adaptive response of the output. Simulations further show that varying the abundance of the E3 ligase strongly influence the output expectedly but does not alter the peak time in motif 1 (Figure 5a), while this is not the case for motif 3 (Figure 5b) where more abundant E3 effectively shifts the output peak time to the left. The models also allow predictions of the steady-state dose–response curves. Interestingly, we see that the steady-state level of the output of motif 1 decreases exponentially with increasing kinase abundance, whereas this output linearly increases for motif 2 (Figure 5c). Thus, augmenting the kinase abundance has opposite regulatory outcomes over the steady-state output levels in these two motifs (Figure 5c). Increasing the E3 ligase abundance leads to a consistent decrease of the output level in all three motifs (Figure 5d). Interestingly, the E3-output dependence curves are pushed lower and become more nonlinear (Figure 5d, dashed lines) when the difference between the ubiquitination rates of S* and pS* becomes more significant due to the kinase, i.e. phosphorylation is more pronounced in influencing ubiquitination.

#### Phosphorylation-mediated ubiquitination motif with feedback

**Figure 6:**
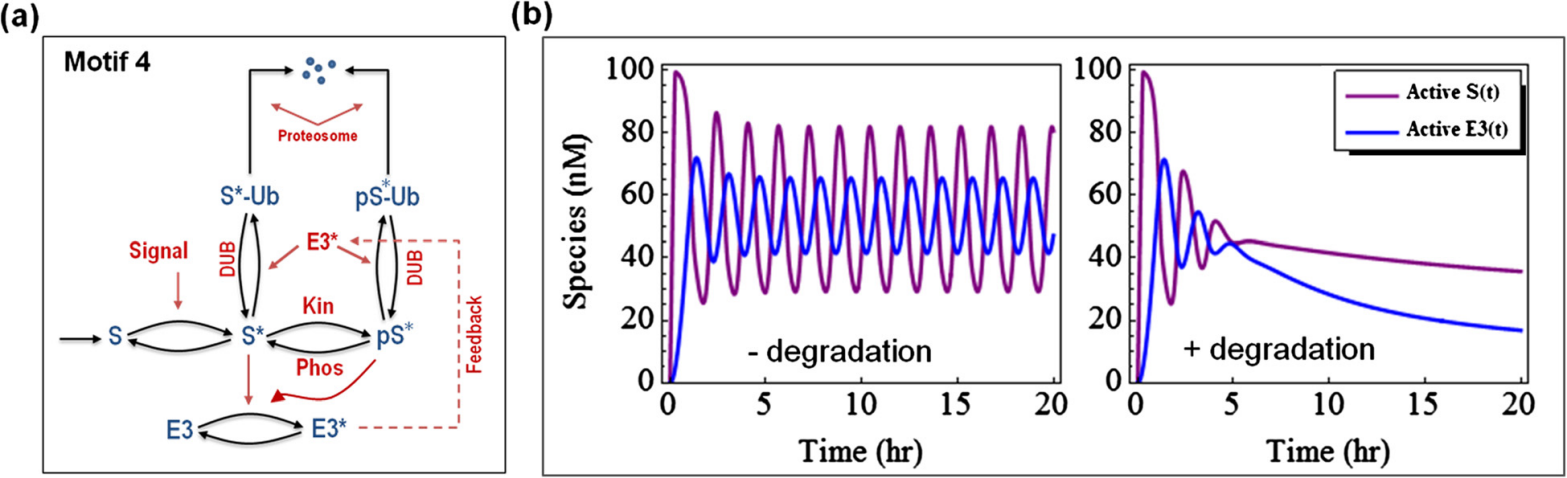
**Schematic diagram and simulations of network motif 4. (a)** Motif 4’s interaction scheme. **(b)** Sustained oscillations for total active S (S* + pS*) and active E3 when the ubiquitinated S moieties are negligibly degraded (left), and damped oscillations when the degradation becomes non-negligible (right). Parameters used are given in the SI.

Feedback loops controlling signalling pathways are commonly seen in ubiquitination-phosphorylation networks. Here, we assume that the output target in motif 1 is an E3 ligase, which can ubiquitinate S* and pS* (displayed in Figure 6a, as motif 4). This creates a negative feedback loop, because an increase in S* will increase the production of active E3 (E3*), which in turn will increase the amount of ubiquitinated S*-Ub and pS*-Ub, which subsequently will decrease the amount of S* and pS*, and thus their output E3*. For protein modification cascades, such as MAPK cascades, it was theoretically predicted [14] and subsequently shown experimentally [15,16,103] that a negative feedback loop can bring about sustained oscillations in the protein activities. These oscillations are caused by the time delay within the negative feedback loop and they also require some degree of ultrasensitivity of individual cascade cycles [14]. Simulations of our ubiquitination-phosphorylation cascade model demonstrate that above certain threshold strength of negative feedback, motif 4 displays sustained oscillations of network species, e.g. active E3 (E3*) or active S (S* + pS*) (shown in Figure 6b). Furthermore, such oscillations can be abolished if S is strongly degraded by the proteosome (Figure 6b, right panel), suggesting the degradation rate can play a determining role in controlling oscillations.

#### Phosphorylation-induced self-ubiquitination

**Figure 7:**
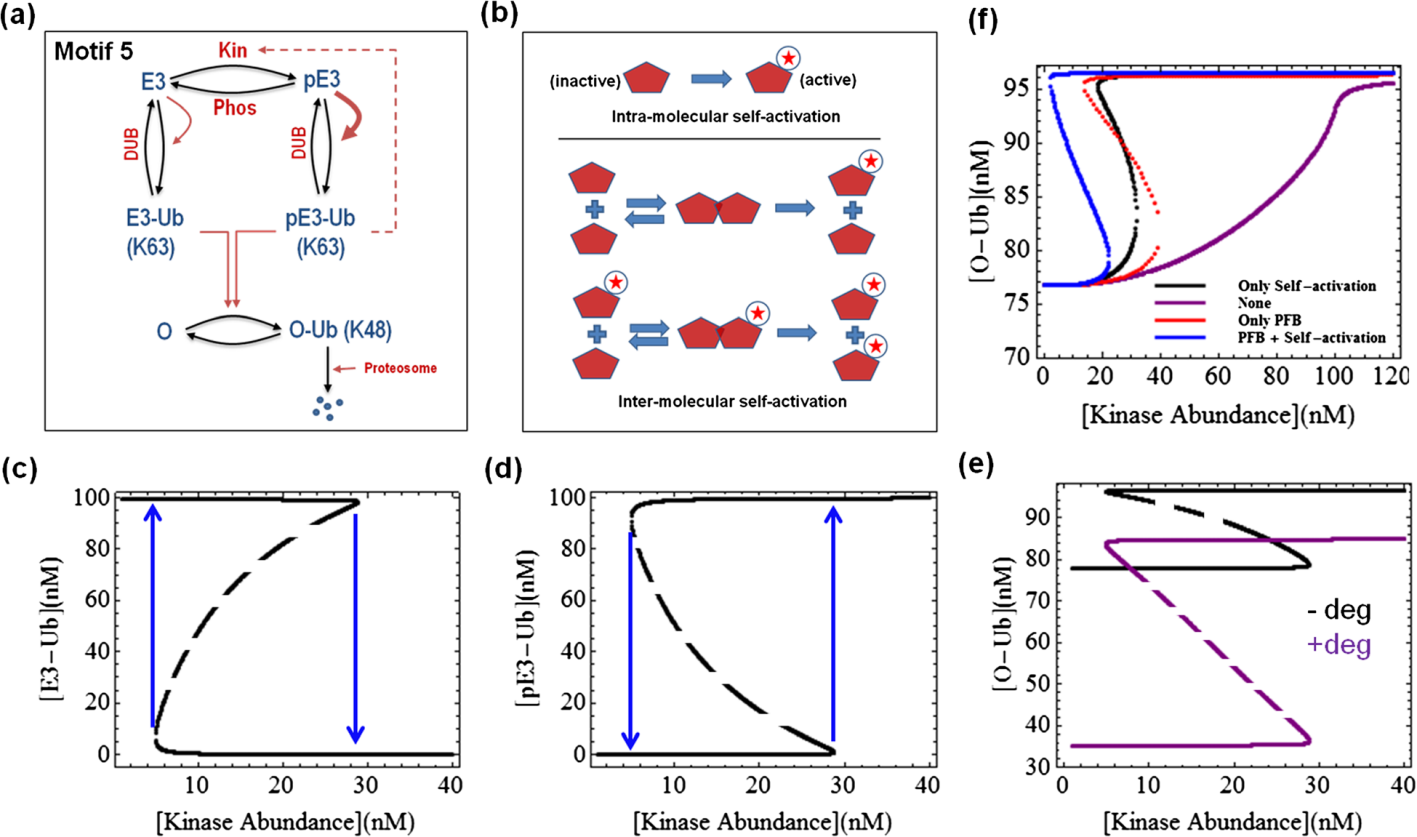
**Schematic kinetic diagram of motif 5 and model simulations. (a)** Dashed line indicates a positive feedback loop from pE3-Ub to the phosphorylation of E3. **(b)** Reaction schemes depicting intra- and inter-molecular self-activation mechanisms. **(c-e)** Steady-state bistable responses of relevant species against gradual increasing of the kinase abundance level. The vertical arrows (blue) indicate the jump between the low and high branch of the hysteresis curves, the dashed lines indicates unstable state. **(f)** Comparison of bistable behaviour under four scenarios when none, only self-ubiquitination, only positive feedback loop, or both mechanisms are operating. Parameters used are given in the SI.

Self-ubiquitination is often observed among the E3 ligases. While often it is a mechanism to self-control the ligase abundance, it can also serve non-proteolytic functions and can dramatically influence the ligase activity, as in the case of ITCH discussed earlier. Degradation of ITCH is independent of its self-ubiquitination, which occurs through K63 linkages and results in stronger catalytic activity; whereas canonical K48-linked chains generated by other ligases target ITCH for degradation [83]. Likewise, self-ubiquitination of NEDD4 leads to better recognition and higher rate of monoubiquitination of Eps15 in the EGFR internalisation and degradation pathway [32]. Other E3 ligases with similar property include Ring1B (component of the human Polycomb transcriptional Repressive Complex 1) whose self-ubiquitination generates atypical, branched K6/K27-linked chains and promotes its monoubiquitination activity toward histone H2A [3,7]. Motivated by these examples, we next analyse a motif where kinase-mediated phosphorylation enhances the rate of self-ubiquitination of an E3 ligase on K63 linkages, which subsequently turns on its ligase activity towards a substrate O, sending it to degradation (Figure 7a, motif 5). Note that, in some cases, the K63-ubiquitinated E3 can directly or indirectly exert positive regulation over the kinase, providing a positive feedback to the system. We will first consider motif 5 with no feedback.

Self-modification reactions can occur in either an intra-molecular or inter-molecular fashion, as depicted in Figure 7b. While our modelling analysis shows that the intra-molecular self-ubiquitination of the E3 ligase does not exhibit intricate dynamics, an inter-molecular form of self-ubiquitination, such as of ITCH discussed above, can bring about bistable behaviour to the system, even without the positive feedback loop [3,13]. Figures 7c,d show bistability and hysteresis for the ubiquitinated forms of the ligase in response to the kinase abundance changes. Interestingly, E3-Ub and pE3-Ub have opposing off and on switches with the increasing kinase level. Similarly, the output also shows a bistable response, with the hysteresis curve being lower in the presence of high degradation rate (Figure 7e). Finally, we analyse motif 5 when the E3-to-Kinase positive feedback loop is also incorporated. Model analysis reveals that although self-ubiquitination or positive feedback alone is sufficient to give rise to bistability, adding the positive feedback appears to enhance self ubiquitination-induced bistability while adding self-ubiquitination does not necessarily enhance bistability established by the positive feedback (comparing blue to black curves, and blue to red in Figure 7f). Moreover, Figure 7f shows that the presence of both mechanisms brings the systems closer to irreversible hysteresis, indicated by the shift to the left of the corresponding hysteresis curve (blue line).

As discussed earlier, ubiquitination is a multi-step process which depends not only on the abundance and properties of the E3 ligase involved but also on other factors involving the preceding steps, including loading of ubiquitin onto conjugating enzymes E2s and ubiquitin transferring to the substrate. Consideration of these factors may be necessary for a detailed model of the control of the EGFR pathway by ubiquitination. Such work however would require comprehensive experimental effort to provide the missing kinetic data and other quantitative information to calibrate and validate the model.

Our findings of potentially bistable and oscillatory behaviour of the ubiquitination-phosphorylation motifs await experimental testing. *In vitro* experimental design based on the model analysis results could be the first step in confirming the predictions about the dynamics of the components of interest. An advantage of an *in vitro* system with purified forms of relevant E3 ligase, kinases and phosphatases is that it can be used to explore wide ranges of precisely set enzyme concentrations. To mimic the *in vivo* situation, some of these proteins may be embedded into a phospholipid membrane bilayer or liposomes if required, which can also facilitate the formation of protein complexes and increase reaction rates [104]. For instance to detect oscillations in motif 4, the system can be started by addition of the relevant input signal, followed by addition of ubiquitin, the E1/E2 enzymes, E3 ligase, kinase and ATP to the reaction medium. At periodic selected time points, aliquots are taken, and the phosphorylated or ubiquitinated level of the substrate can be measured by immunoblotting using specific antibodies for phosphorylation or ubiquitination. It is however worth mentioning that assembling an oscillatory network *in vitro* is challenging due to a multitude of factors at play, including the adequate level of ubiquitin and the essential participation of the relevant E1/E2 enzymes. Therefore, direct *in vivo* approaches like imaging techniques using microscopy-based binding assay can be exploited for high temporal resolution measurements of components kinetics and may be a more favourable option [105]. On the other hand, detection of switches such as in motif 5 can be done by similar measurement techniques in response to increasing titration of a dose component, in this case the involved kinase protein (Figure 7).

In summary, we have constructed mathematical models and carried out analysis for a number of commonly seen motifs of ubiquitination-phosphorylation crosstalk. The motifs, although simplified, show diverse dynamics including sustained oscillations and bistability. More importantly, the models have facilitated the identification of the conditions under which these dynamics may realise, which would have been infeasible if such models are not used. Modelling therefore provides a useful and necessary tool for efficient analysis of ubiquitination-phosphorylation crosstalk, thereby potentially improving our systems-level understanding of the integrated EGFR signalling.

## Conclusions

Since the first discovery of protein ubiquitination more than three decades ago, extensive work has revolutionized our perception of its role in signalling networks. Not only protein ubiquitination serves as a main mechanism for protein degradation, emerging evidence has revealed that different types of ubiquitin chains can induce a variety of non-proteolytic functions and can dramatically alter the biological activities of a target protein. On top of that, ubiquitination is frequently observed to interplay with other PTMs such as phosphorylation or sumoylation to coordinate regulation of signalling processes in intricate manners. Such complexity arising from interconnected PTM networks poses enormous challenges for the systems level analysis of signalling processes. Mathematical modelling is emerging as a valuable tool to provide insight into their dynamic behaviour that would otherwise not be possible. Mathematical models help combine the mechanistic, molecular knowledge with rigorous analysis of the complex output dynamics of the PTM networks.

The expanding roles of ubiquitylation and phosphorylation in cell signalling, to large extent, have been uncovered thanks to recent advances in proteomics technologies which have enabled new ways for in-depth, unbiased and quantitative analysis of different PTMs on a global scale [106-110]. Techniques such as stable isotope labelling with amino acids in cell culture (SILAC) and label-free based mass spectrometry can quantify changes in expression of thousands of phosphoproteins and tens of thousands phosphorylation events in a single experiment and have become well established [106,111]. Although proteome-wide analysis of endogenous ubiquitination has been more challenging, recent developments on antibodies-based enrichment methods demonstrate the feasibilities of similar large-scale, quantitative and site-specific investigations of this PTM [112]. Moreover, novel methods that are aimed at identifying proteins comodified by both phosphorylation and ubiquitination have revealed exciting global details of the cross-regulation between these two PTMs [113]. A major limitation with current mass spectrometry based methods however is the inability to distinguish among modifications by ubiquitination, NEDD8 or ISG15, due to an identical di-Gly remnant generated by trypsin proteolysis of the modified proteins [112]. Nevertheless, it is likely that with the observed fast pace of technological advance, sophisticated methods capable of resolving at even higher quantitative resolution the extent of PTMs crosstalk and their distinct dynamics under different cellular perturbations are within close reach. These data will undoubtedly be valuable inputs to the construction of large-scale, next-level quantitative models. A global, data-driven modelling-based understanding of PTMs networks and the ability to simulate their behaviour and form testable predictions will open countless possibilities that can drive the frontiers of both biological and medical research.

## Abbreviations

DUB: De-ubiquitinating enzyme; EGF: Epidermal growth factor; EGFR: Epidermal growth factor receptor; ERK: Extracellular signal-regulated kinase; Cbl: Casitas b-lineage lymphoma; RTK: Receptor tyrosine kinase; EPS15: Epidermal growth factor receptor substrate 15; MVB: Multivesicular body; UIM: Ubiquitin-interacting motif; HRS: Hepatocyte growth factor-regulated tyrosine kinase substrate; HECT: Homologous to the E6-AP carboxyl terminus; STAMP: Signal transducing adaptor molecule; STAMBP: STAM binding protein; USP8: Ubiquitin specific peptidase 8; Rab5: Ras-related protein Rab5; GEF: Guanine nucleotide exchange factor; GA: Benzoquinone ansamycin Geldanamycin; JNK: c-Jun N-terminal kinase; MEKK1: MEK kinase 1; ITCH Itchy: E3 ubiquitin protein ligase; NEDD4: Neural precursor cell expressed developmentally down-regulated protein 4; Rpn4: Regulatory particle non-ATPase; RING1B: Really interesting new gene 1 protein.

## Competing interests

The authors declare that they have no competing interests.

## Authors’ contributions

LKN and BK designed the project. LKN carried out model simulations and analysis. LKN, WK and BK wrote the paper. All authors read and approved the final manuscript.

## Supplementary Material

Additional file 1 Mathematical models for the investigated motifs. Click here for file
