# Supplementary material for: When ubiquitination meets phosphorylation: a systems biology perspective of EGFR/MAPK signalling
Source: Cell Commun Signal. 2013 Jul 31;11:52. doi: 10.1186/1478-811X-11-52 (PMC3734146; doi:10.1186/1478-811X-11-52)
Supplement: Additional file 1 — Mathematical models for the investigated motifs. [file 1478-811X-11-52-S1.docx]

**Supplemental Information**

**When ubiquitination meets phosphorylation: a systems biology perspective of EGFR/MAPK signalling**Lan K. Nguyen^1,^*, Walter Kolch^1,2^, Boris N. Kholodenko^1,2,^*

^1^ Systems Biology Ireland, University College Dublin, Dublin 4, Ireland

^2^ Conway Institute of Biomolecular & Biomedical Research,

University College Dublin, Dublin 4, Ireland

* Corresponding authors: [lan.nguyen@ucd.ie](mailto:lan.nguyen@ucd.ie); boris.kholodenko@ucd.ie

**Mathematical Models for the investigated Motifs**

In this supplementary information, we present in details the reactions, parameter values, and ordinary differential equations for the models of all motifs investigated in the main text.

**Motif 1 and 2:**

**Figure S1. Kinetic schemes of the Motif 1 and 2 analysed in the main text, with reactions numbered.** The two motifs differ only in the magnitude of the E3-mediated ubiquitination rate of S* and pS, as indicated by the thickness of the highlighted blue arrows (thicker line means stronger rate).


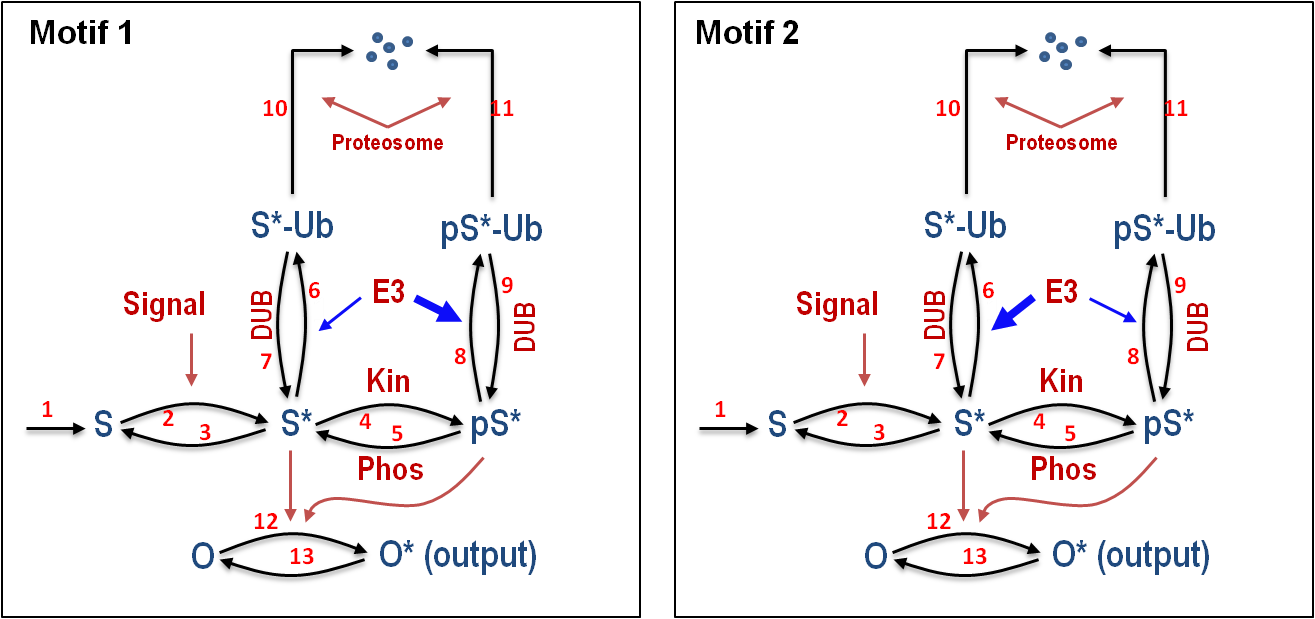


**Table S1. Reactions and reaction rates of the kinetic model for Motif 1.** Concentrations and the Michaelis-Menten constants (K_m_s) are given in nM. First- and second-order rate constants are expressed in s^-1^ and nM^-1^ s^-1^. Maximum rates Vs are expressed in nM s^-1^*.*

| **Reaction number** | **Reactions** | **Reaction rates** | **Parameter values** |
| --- | --- | --- | --- |
| **1** | Ø → S | **$\text{v}_{1}\text{= k}_{1}$** | k_1_= 0.0001 |
| **2** | S → S* | **$\text{v}_{2}\text{= }\frac{\text{k}_{2}\cdot\text{Signal}\cdot\text{S(t)}}{\text{K}_{\text{m2}}+\text{S(t)}}$** | k_2_= 0.001, K_m2_=100 Signal = 10 |
| **3** | S* → S | **** | V_3_= 0.01,  K_m3_=100 |
| **4** | S* → pS* | **** | k_4_= 0.001, K_m4_=100 Kin = 100 |
| **5** | pS* → S* | **** | k_5_= 0.005, K_m5_=100 Phos = 100 |
| **6** | S* → S*-Ub | **** | k_6_= 0.001(*), K_m6_=100 E3 = 100 |
| **7** | S*-Ub → S* | **** | k_7_= 0.01, K_m5_=100 DUB = 100 |
| **8** | pS* → pS*-Ub | **** | k_8_= 0.1 (**), K_m8_=100 E3 = 100 |
| **9** | pS*-Ub → pS* | **** | k_9_= 0.001, K_m9_=100 DUB = 100 |
| **10** | S*-Ub → Ø | **** | k_10_= 0.1 |
| **11** | pS*-Ub → Ø | **** | k_11_= 0.1 |
| **12** | O → O* | **** | k_12a_= k_12b_ =0.001,  K_m12_=100 |
| **13** | O* → O | **** | V_13_= 0.01,  K_m13_=100 |

(*,**) Note that everything is the same for the model of Motif 2, except we have:
k_6_= 0.01 and k_8_= 0.001 instead to reflect the weaker ubiquitination rate of the phosphorylated pS* compared to that of S*.

**Table S2. Ordinary differential equations of the kinetic model for Motif 1 and 2.** The reaction rates are given in Table S1.

| **Left-hand Sides** | **Right-hand Sides** | **Initial Concentrations (nM)** |
| --- | --- | --- |
| d[S]/dt | v_1_ –v_2_ + v_3_ | 100 |
| d[S*]/dt | v_2_ – v_3_ – v_4_ + v_5_  –v_6_ + v_7_ | 0 |
| d[pS]/dt | v_4_ – v_5_ – v_8_ + v_9_ | 0 |
| d[S*-Ub]/dt | v_6_ –v_7_ – v_10_ | 0 |
| d[pS-Ub]/dt | v_8_ – v_9_ – v_11_ | 0 |
| d[O]/dt | –v_12_ + v_13_ | 100 |
| d[O*]/dt | v_12_ –v_13_ | 0 |

**Motif 3:**


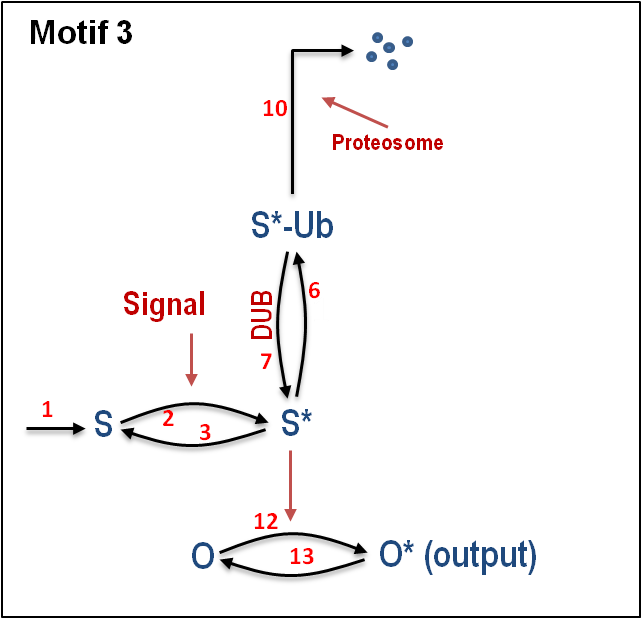


**Figure S2. Kinetic schemes of the Motif 3 analysed in the main text, with reactions numbered.** Here the ubiquitination-triggered degradation of the active protein S*is not dependent on phosphorylation. For convenience, we retain the same reaction numbers as in Motif 1, 2 for the remaining reactions.

**Table S3. Ordinary differential equations of the kinetic model for Motif 3.** The reaction rates are given in Table S1.

| **Left-hand Sides** | **Right-hand Sides** | **Initial Concentrations (nM)** |
| --- | --- | --- |
| d[S]/dt | v_1_ –v_2_ + v_3_ | 100 |
| d[S*]/dt | v_2_ – v_3_ – v_6_ + v_7_ | 0 |
| d[S*-Ub]/dt | v_6_ –v_7_ – v_10_ | 0 |
| d[O]/dt | –v_12_ + v_13_ | 100 |
| d[O*]/dt | v_12_ –v_13_ | 0 |

**Motif 4:**


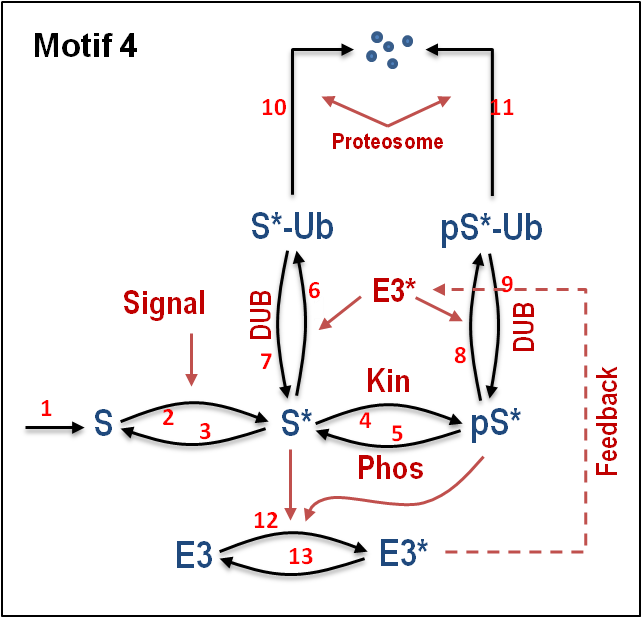


**Figure S3. Kinetic schemes of the Motif 4 analysed in the main text, with reactions numbered.** In this case, the motif output is the active form of the E3 ligase, which subsequently forms a negative feedback to the upstream protein S*.

**Table S4. Reactions and reaction rates of the kinetic model for Motif 4.** Concentrations and the Michaelis-Menten constants (K_m_s) are given in nM. First- and second-order rate constants are expressed in s^-1^ and nM^-1^ s^-1^. Maximum rates Vs are expressed in nM s^-1^*.*This parameter set was used to simulate the oscillatory dynamics as shown in Fig.3 in the main text.

| **Reaction number** | **Reactions** | **Reaction rates** | **Parameter values** |
| --- | --- | --- | --- |
| **1** | Ø → S | **$\text{v}_{1}\text{= k}_{1}$** | k_1_= 0.00005 |
| **2** | S → S* | **$\text{v}_{2}\text{= }\frac{\text{k}_{2}\cdot\text{Signal}\cdot\text{S(t)}}{\text{K}_{\text{m2}}+\text{S(t)}}$** | k_2_= 0.0025, K_m2_=3.35 Signal = 50 |
| **3** | S* → S | **** | V_3_= 0.0083,  K_m3_=25 |
| **4** | S* → pS* | **** | k_4_= 0.0005, K_m4_=50 Kin = 100 |
| **5** | pS* → S* | **** | k_5_= 0.0004, K_m5_=50 Phos = 100 |
| **6** | S* → S*-Ub | **** | k_6_= 0.0045, K_m6_=1 E3 = 100 |
| **7** | S*-Ub → S* | **** | k_7_= 0.0025, K_m5_=5 DUB = 100 |
| **8** | pS* → pS*-Ub | **** | k_8_= 0.00002 , K_m8_=10 E3 = 100 |
| **9** | pS*-Ub → pS* | **** | k_9_= 0.007, K_m9_=30 DUB = 100 |
| **10** | S*-Ub → Ø | **** | k_10_= 0.000001 |
| **11** | pS*-Ub → Ø | **** | k_11_= 0.0001 |
| **12** | E3 → E3* | **** | k_12a_= 0.00001, k_12b_ =0.0016,  K_m12a_=20, K_m12b_=5, |
| **13** | E3* → E3 | **** | V_13_= 0.05,  K_m13_=10 |

**Table S5. Ordinary differential equations of the kinetic model for Motif 4.** The reaction rates are given in Table S4.

| **Left-hand Sides** | **Right-hand Sides** | **Initial Concentrations (nM)** |
| --- | --- | --- |
| d[S]/dt | v_1_ –v_2_ + v_3_ | 100 |
| d[S*]/dt | v_2_ – v_3_ – v_4_ + v_5_  –v_6_ + v_7_ | 0 |
| d[pS]/dt | v_4_ – v_5_ – v_8_ + v_9_ | 0 |
| d[S*-Ub]/dt | v_6_ –v_7_ – v_10_ | 0 |
| d[pS-Ub]/dt | v_8_ – v_9_ – v_11_ | 0 |
| d[E3]/dt | –v_12_ + v_13_ | 100 |
| d[E3*]/dt | v_12_ –v_13_ | 0 |

**Motif 5:**


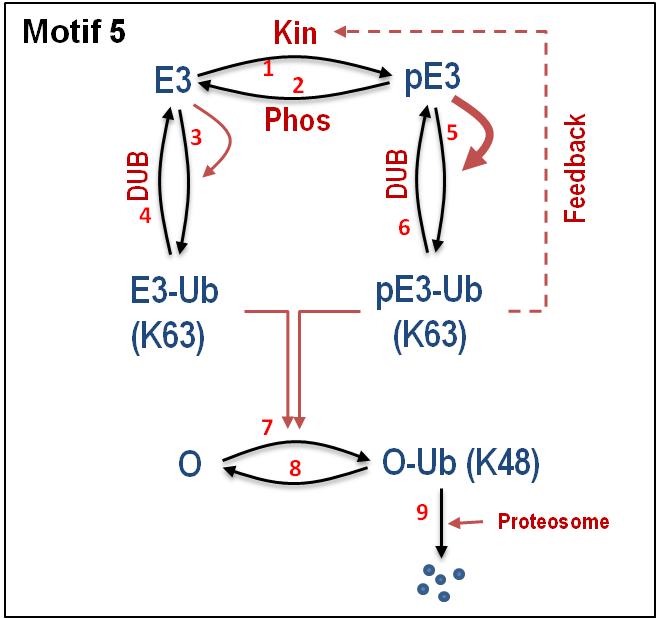


**Figure S4. Kinetic schemes of the Motif 5 analysed in the main text, with reactions numbered.** We consider two cases when a positive feedback from pE3-Ub to the Kinase is present (dashed line) or absent.

**Table S6. Reactions and reaction rates of the kinetic model for Motif 5.** Concentrations and the Michaelis-Menten constants (K_m_s) are given in nM. First- and second-order rate constants are expressed in s^-1^ and nM^-1^ s^-1^. Maximum rates Vs are expressed in nM s^-1^*.*This parameter set was used to simulate bistable dynamics as shown in Figure 4 of the main text.

| **Reaction number** | **Reactions** | **Reaction rates** | **Parameter values** |
| --- | --- | --- | --- |
| **1** | E3 → pE3 | **$\text{v}_{1}\text{= }\frac{\text{k}_{1}\cdot\text{Kin}\cdot\text{E}3\text{(t)}}{\text{K}_{\text{m1}}+\text{E}3\text{(t)}}\cdot\left(1+\text{k}_{f}\cdot\text{pE}3\text{-Ub(t)}\right)$** | k_1_= 0.01 k*_f_*= 0 (no feedback) k*_f_*= 0.05 (feedback) Kin =100 |
| **2** | pE3 → E3 | **$\text{v}_{2}\text{= }\frac{\text{k}_{2}\cdot\text{Phos}\cdot\text{pE}3\text{(t)}}{\text{K}_{\text{m2}}+\text{pE}3\text{(t)}}$** | k_2_= 0.01, K_m2_=100  Phos =100 |
| **3** | E3 → E3-Ub | **** | k_3_= k_3a_ = 0.01 |
| **4** | E3-Ub → E3 | **$\text{v}_{4}\text{= }\frac{\text{k}_{4}\cdot\text{DUB}\cdot\text{E}3\text{-Ub(t)}}{\text{K}_{\text{m4}}+\text{E}3\text{-Ub(t)}}$** | k_4_= 0.01, K_m4_=50 DUB = 100 |
| **5** | pE3 → pE3-Ub | **$\text{v}_{5}\text{= pE}3\text{(t)}\cdot\left(\text{k}_{5}\cdot\text{pE}3\text{(t)+k}_{5\text{a}}\cdot\text{pE}3\text{-Ub(t)}\right)$** | k_5_= 0.01, k_5a_ = 0.1 |
| **6** | pE3-Ub → pE3 | **$\text{v}_{6}\text{= }\frac{\text{k}_{6}\cdot\text{DUB}\cdot\text{pE}3\text{-Ub(t)}}{\text{K}_{\text{m6}}+\text{pE}3\text{-Ub(t)}}$** | k_6_= 0.01, K_m6_=50 |
| **7** | O → O-Ub | **$\text{v}_{7}\text{= }\frac{\text{k}_{7}\cdot\text{E}3\text{-Ub(t)}\cdot\text{O(t)}}{\text{K}_{\text{m7}}+\text{O(t)}}+\frac{\text{k}_{\text{7a}}\cdot\text{pE}3\text{-Ub(t)}\cdot\text{O(t)}}{\text{K}_{\text{m7}}+\text{O(t)}}$** | k_7_= 0.01, k_7a_ = 0.01, K_m7_=50 |
| **8** | O-Ub → O | **$\text{v}_{8}\text{= }\frac{\text{V}_{8}\cdot\text{O-Ub}(\text{t})}{\text{K}_{\text{m8}}+\text{O-Ub}(\text{t})}$** | V_8_= 0.5,  K_m8_=50 |
| **9** | O-Ub → Ø | **$\text{v}_{9}\text{= k}_{9}\cdot\text{O-Ub}(\text{t})$** | k_9_= 0 (for no degradation, 0.01 for degradation) |

(*) For derivation of the kinetic expression for auto-ubiquitination, see (Nguyen et al., 2011)

**Table S7. Ordinary differential equations of the kinetic model for Motif 5.** The reaction rates are given in Table S6.

| **Left-hand Sides** | **Right-hand Sides** | **Initial Concentrations (nM)** |
| --- | --- | --- |
| d[E3]/dt | –v_1_ +v_2_ – v_3_ + v_4_ | 100 |
| d[pE3]/dt | v_1_ –v_2_ – v_5_ + v_6_ | 0 |
| d[E3-Ub]/dt | v_3_ – v_4_ | 0 |
| d[pE3-Ub]/dt | v_5_ – v_6_ | 0 |
| d[O]/dt | v_8_ – v_7_ | 100 |
| d[O-Ub]/dt | v_7_ – v_8_ | 0 |

**Reference:**

Nguyen, L. K., Munoz-Garcia, J., Maccario, H., Ciechanover, A., Kolch, W., & Kholodenko, B. N. (2011). Switches, excitable responses and oscillations in the Ring1B/Bmi1 ubiquitination system. *PLoS Comput Biol, 7*(12), e1002317.
